# Rapid Sequence Induction on trauma patients performed by a rural and suburban air ambulance service: a 16-month audit of practice

**DOI:** 10.1186/1757-7241-21-S1-S11

**Published:** 2013-05-28

**Authors:** A Chesters, N Keefe, J Mauger

**Affiliations:** 1 East Anglian Air Ambulance, UK

## Introduction

This poster describes the first 16 months experience of pre-hospital RSI in a rural and sub-urban helicopter-based doctor-paramedic service after the introduction of a standard operating procedure already proven in an urban trauma environment.

## Method

A retrospective database review of all missions between October 2010 and January 2012 was carried out. Any RSI or intubation carried out on an injured patient was included, regardless of age or indication. Patients who were intubated by Ambulance Service personnel prior to the arrival of the EAAA team were excluded.

## Results

The team was activated 1156 times and attended 763 cases. A total of 69 RSIs occurring within the study period were identified as having been carried out by the EAAA team. There were no failed intubations that required a rescue surgical airway or the placement of a supraglottic airway device. For RTCs, the overall on scene time for patients who required an RSI was 40 minutes (range 15-72 minutes). For all other trauma, the average on scene time was 48 minutes (range 25-77 minutes).

## Conclusion

We have demonstrated the successful introduction of a pre-hospital care standard operating procedure, already tested in the urban trauma environment, to a rural and suburban air ambulance service operating a full-time doctor-paramedic model. We have shown a zero failed intubation rate over 16 months of practice during which time over 750 missions were flown with 11.5% of these resulting in an RSI.

